# The law of food allergy and accommodation in Canadian schools

**DOI:** 10.1186/s13223-018-0273-6

**Published:** 2018-10-22

**Authors:** Blake Murdoch, Eric M. Adams, Timothy Caulfield

**Affiliations:** Faculty of Law, Law Centre, University of Alberta, Edmonton, AB T6G 2H5 Canada

**Keywords:** Allergy policy, Health law, Anaphylaxis, School policy, Human rights, Disability

## Abstract

**Background:**

There is ongoing controversy surrounding the appropriate standards and limits of accommodation of children with food allergies in schools. We identify and explain how relevant Canadian common law, legislation, constitutional law and human rights policy can inform future school policy around allergy, disability and food bans.

**Main body:**

The *Canadian Charter of Rights and Freedoms* applies to governmental laws or policies, including the policies of schools, and grants every individual the right to freedom from discrimination based on, among other things, disability. Canadian constitutional and human rights law define disability broadly including perceived disabilities. Provincial human rights tribunals in both Ontario and BC have explicitly found allergy to be a disability requiring accommodation, even in cases not involving anaphylaxis risk. However, the cases most pertinent to the scenarios faced by schools have found that food bans may not be required, due to recent scientific evidence that they do not render allergy sufferers safer.

**Conclusion:**

Anaphylaxis-level allergy constitutes a disability under both the *Charter* and human rights legislation, despite the fact that higher courts have not definitively ruled on the matter. Accordingly, schools must make careful decisions about how to deal with life-threatening allergies among their students. Food bans are generally not legally necessary, and, in the absence of new legislation, are only likely to become so if sufficient scientific evidence demonstrates that they increase safety for students. School policies should be substantially informed by evidence-based research in order to ensure ongoing congruence with human rights law.

## Background

Allergy represents a significant and growing public health challenge in Canada and worldwide [1]. Indeed, the prevalence of food allergy seems to be increasing [2, 3], and children are the most likely demographic group to develop allergies [4]. Allergies affect over a third of the world’s adolescent population [5, 6]. Given the prevalence of childhood allergy, the question of how we treat food allergies in schools—a location in which children tend to experience frequent exposure to food products—is of key importance to overall food allergy policy. There is a need to better understand the legal and policy foundation of school allergy guidelines, and to answer the question of whether and when allergy should be treated as a legal disability. Our goal is to identify and explain how relevant Canadian common law, legislation, constitutional law and human rights policy can inform future school policy around allergy, disability and food bans.

## Canadian context

In Canada, uncertainty exists among parents, educators and policymakers regarding the treatment of food allergy in schools [7]. Despite a range of relevant allergy policies and provincial regulation, conflict and concern continue about how to balance the interests of individual children who suffer from allergies with the interests of institutions, other children, and the logistics and inconveniences of navigating multiple food bans in the classroom [8]. Moreover, some medical professionals have pointed out that food bans may fail to achieve the outcomes they seek, as evidence shows that the rate of accidental exposure to, for example, peanuts in peanut-free schools “is not lower, compared to those that do not prohibit peanuts” [9, 10]. A 2015 Canadian study that tracked 567 anaphylactic events found no statistically significant difference in the percentage of events that occurred in schools or daycares prohibiting peanuts and those that allowed them [11]. Such results lead some to question the necessity of, or even the medical basis for food bans in schools beyond preschool age [12].

Some of the tension about school allergy policy might be related to a perception that not everyone who claims to have a food allergy actually does [13]. Indeed, a 2017 study found that the prevalence of food allergies was less than previously estimated [14]—a conclusion that received international media attention [15]. Another 2017 study came to a similar conclusion regarding tree nut allergies [16], emphasizing that food allergies might be over-diagnosed [17, 18]. Recent research also suggests inappropriate or unnecessary allergy testing may be undertaken too frequently, leading to over-diagnosis of allergy. A consensus report by the National Academies of Sciences, Engineering and Medicine stated that “physicians should not order ‘panels’ of food tests without a rationale,” especially since many tests are far from definitive [13]. Beyond testing provided by physicians, complementary and alternative medicine practitioners, such as naturopaths, are now marketing services to diagnose and assess food “sensitivities” [19], often using methods like food-specific immunoglobulin G testing which have been widely discredited by allergists and the scientific community [20]. Media reports about controversial issues like prevalence and the efficacy of popular testing strategies seem likely to complicate public understandings of the medical dynamics of allergy.

Studies confirm a significant amount of public misunderstanding surrounding allergies and the associated risks, particularly in the context of children [21, 22]. It is currently very difficult to accurately predict whether an individual will develop anaphylaxis [23], and clinical diagnosis is currently based primarily on clinical history of exposure to triggering agents [24]. While much focus is placed on peanuts as agents causing anaphylaxis, shellfish, tree nuts, eggs and milk can also give rise to the condition with significant frequency [25, 26]. These realities, along with the conflicting and sometimes inaccurate portrayals of allergy presented by the media and marketing, may contribute to fear and misunderstanding among parents and policy makers.

Jurisdictions across Canada have school allergy policies [27]. These range from individual school board policy to provincial legislation. In 2006, Ontario passed *Sabrina’s Law*, after a 13-year old, Sabrina Shannon, died of an anaphylactic reaction while in school. The law requires school boards to maintain policies and procedures to address the potential for anaphylactic allergic reactions in schools, including strategies for reducing exposure risk, training for employees and communication and action plans [27, 28]. The onus is placed on parents/guardians and pupils to ensure pupil information is up-to-date, and school employees are exempted from actions for damages if, in good faith, they administer an epinephrine injection when given reason to believe a pupil is experiencing anaphylaxis [28]. Laws and policies similar to Sabrina’s Law have been enacted across Canada [29–33].

Given the increasing profile of food allergy issues and, at the same time, controversies about key issues like prevalence, testing and the effectiveness of existing policies, it is not surprising that in recent years parents have challenged schools regarding the treatment of children with severe allergies. The most prominent recent Canadian case is that of Lynne Glover. In 2014, Ms. Glover filed a human rights complaint against her 6 year old daughter’s elementary school, claiming it discriminated against her child for “failing to accommodate her life-threatening allergy to eggs and dairy” [34, 35]. The case was partially settled in August 2014, and the school subsequently prohibited eggs and dairy except for in a special breakfast room [36]. The Ontario Human Rights Commission (OHRC) “Policy on ableism and discrimination based on disability” now specifically mentions that employers, schools and other providers must develop a comprehensive allergy strategy, though it notes that “in some cases, the law is still not clear as to whether certain conditions are disabilities within the meaning of the code” [37, 38].

## The law

As noted by the OHRC, the law of allergy as it relates to children’s rights and the potential classification of allergy as a disability remains unsettled. Confusion has even spread into the realm of criminal law. In 2016, a waiter in Quebec was arrested for mistakenly serving salmon tartare to a customer with severe seafood allergies, causing anaphylactic shock and a 5 days coma [39]. The police recommended criminal charges but, in the end, the Crown prosecutor did not proceed with the case, making the discretionary determination that there was no recklessness but merely an ordinary mistake made in good faith [39]. Provincial human rights tribunal (HRT) proceedings dealing with complaints based on allergy often settle without definitive rulings that would help inform policy. Moreover, public discourse and marketing can confuse legal and policy debates by casting doubt on the definition of a food allergy and the severity of the problem. The popular press, complementary and alternative medicine practitioners, and food industry marketing have contributed to beliefs regarding the widespread existence of “sensitivities” to many foods [19, 40, 41], and social media has enabled the spread of misinformation about allergy [42], such as the false notion that the mere smell of peanut can cause anaphylaxis [43]. Given these confounding factors, we seek to clarify the current legal and regulatory framework.

### International law

In 2010 the Canadian government ratified the United Nations’ convention on the rights of persons with disabilities [44], which states that “disability is an evolving concept”, and that “disability results from the interaction between persons with impairments and attitudinal and environmental barriers that hinders their full and effective participation in society on an equal basis with others” [45]. Furthermore, it states that the parties “recognize the right of persons with disabilities to education”, and that the parties shall ensure “persons with disabilities can access an inclusive, quality and free primary education and secondary education on an equal basis with others in the communities in which they live” [45]. Ratification of the convention commits Canada to the application of the rights found therein, and is binding under international law [44].

### Canadian constitutional law

The *Canadian Charter of Rights and Freedoms* (*Charter*) applies to laws passed by federal, provincial, and municipal governments and to the policies enacted by private entities that are either controlled by government or implementing a specific government program or policy. Accordingly, any federal, provincial, or municipal laws or policies dealing with allergy will need to conform with the rights enshrined by the *Charter*. Whether the *Charter* applies directly to the decisions of school boards or individual elementary or secondary schools is somewhat less certain [46]. Nonetheless, it is likely that a court would find that school boards or schools themselves are implementing a government policy when enacting rules dealing with student allergies, and hence are subject to the *Charter* [47].

Section 15 of the *Charter* states that “Every individual is equal before and under the law and has the right to the equal protection and equal benefit of the law without discrimination and, in particular, without discrimination based on race, national or ethnic origin, colour, religion, sex, age or mental or physical disability” [48]. Section 15(2) goes on to state that this equality right “does not preclude any law, program or activity that has as its object the amelioration of conditions of disadvantaged individuals or groups including those that are disadvantaged because of […] mental or physical disability” [48]. This second part may justify food bans that affect non-allergic children for the benefit of others who bear anaphylaxis risk.

The Supreme Court of Canada (SCC) has stressed that the purpose of s. 15(1) “is to ensure equality in the formulation and application of the law. The promotion of equality entails the promotion of a society in which all are secure in the knowledge that they are recognized at law as human beings equally deserving of concern, respect, and consideration” [49]. More recently, the Court has stressed that the “animating norm” of s. 15 is “substantive equality,” the idea that true equality is often achieved not by treating things alike but by recognizing the need for differential treatment when appropriate [50]. An analysis under s. 15(1) involves two determinations: (1) does the law create a distinction based on an enumerated or analogous ground listed in s. 15(1); and (2) does that distinction discriminate by perpetuating disadvantage? [51].

Answering the question of whether or not distinctions discriminate requires examining a number of contextual factors including the nature and purpose of the law or legislative scheme, the circumstances and history of the individual as a member of a group claiming protection under s. 15(1), and the nature of the impact of the law on the rights claimant [50]. Importantly, this test is not focused on whether a discriminatory attitude exists, but rather on assessing and preventing discriminatory impacts [51].

While the *Charter* does not define physical disability, it is likely that allergies constitute protected physical disabilities, given the judicial preference to interpret the *Charter*’s terms “generously and purposively”, in a way “suitable to give individuals the full measure of the fundamental rights and freedoms referred to” [47]. Discussing the nature of disability more broadly, the Court has held that it “means vastly different things depending upon the individual and the context” [52]. It has stated:

The concept of disability must therefore accommodate a multiplicity of impairments, both physical and mental, overlaid on a range of functional limitations, real or perceived, interwoven with recognition that in many important aspects of life the so-called “disabled” individual may not be impaired or limited in any way at all [53].

Accordingly, a determination of disability requires a contextual analysis, taking into account the characteristics of the individual and the surrounding circumstances [54]. The SCC adds in *Granovsky* that, “while the notions of impairment and functional limitation are important considerations in disability analysis” under the *Charter*, the primary focus is on inappropriate legislative or administrative response or inaction in dealing with disadvantage [53]. That is to say, section 15(1) disability analysis is focused primarily on discriminatory treatment, and not on assessing the precise particulars of an individual’s biomedical conditions [53].

Assuming the *Charter* applies to school allergy policies, an argument could be made under section 15(1) that: (1) a severely allergic child (that is, a child deemed to be at increased risk for anaphylaxis) experiences differential treatment by not having equal access to a safe school environment for education on the basis of the allergy; (2) allergy falls under the enumerated ground of physical disability, and; (3) the child was discriminated against because a failure to consider and accommodate the particular circumstances of students afflicted with allergies fails “to ensure that they benefit equally from a service offered to everyone” [47]. As Justice La Forest held in the *Eldridge* case, the historical disadvantage of disabled persons has “been shaped and perpetuated by the notion that disability is an abnormality or flaw,” and the failure to consider what persons with disabilities require in order to access general services to fully participate in society [47].

A court examining a school’s allergy policy (or absence of one) will look at all of the circumstances surrounding the policy and its objectives in order to determine whether or not that policy discriminates against allergy sufferers. In this respect, it will be relevant if schools have created their policies in light of the medical literature on allergy and food bans. For example, a court may hear evidence that food prohibitions are not widely medically recommended (though, we note, they sometimes are [55]) or that some have speculated that they may actually increase safety risks posed to students dealing with allergies because of the false sense of security such bans create. Such evidence suggests that schools may have a strong argument that policies implementing a less than total food prohibition does not discriminate against individuals dealing with a disability caused by allergy [56].

Even if a particular policy is found to discriminate against individuals suffering from allergy, it may still be lawful as a “reasonable limit” on equality rights. Section 1 of the *Charter* states that the rights and freedoms therein are “subject only to such reasonable limits prescribed by law as can be demonstrably justified in a free and democratic society” [48]. Determining whether a breach of s. 15 is justified under section 1 requires analysis under the test developed in *R v Oakes* [57]. Courts will analyze whether the law possesses a “pressing and substantial purpose”, taking into consideration the values essential to a free and democratic society, which include human dignity, social justice, social equality, and others [57]. Additionally, courts must find the law proportional to the achievement of its objective. Assessing proportionality is a three-step process that includes demonstrating: (1) a rational connection between the measures and the objective, (2) minimal impairment of rights and freedoms, and (3) that the overall social benefits achieved by the law outweigh the detriments to the rights holders [57].

A section 1 analysis would weigh the impacts of partial or total food bans on all students. Justifying the reasonability of an allergy policy will include attention to the rights, freedoms, and interests of others engaged by the particular policy at issue [58, 59]. For example, it could be argued that the absence of a total food prohibition promotes vigilance from school officials and students in a way that better protects the well-being of students dealing with allergy.

### Human rights law and policy

Whether or not the *Charter* applies to schools and their allergy policies, it is clear that provincial human rights legislation does. Because education is largely a provincial responsibility, provincial human rights legislation rather than the *Canadian Human Rights Act* governs most school boards, schools, and their policies [60]. The provinces and territories each have human rights legislation protecting against discrimination on the grounds of disability, and most of them, with the exception of British Columbia, Manitoba and Quebec [61–63], specifically define the term. Alberta, Ontario, New Brunswick, and Prince Edward Island all use nearly identical definitions of physical disability, stating that it is “any degree of physical disability, infirmity, malformation or disfigurement that is caused by bodily injury, birth defect or illness and, without limiting the generality of the foregoing, includes epilepsy, paralysis, amputation, lack of physical co-ordination, blindness or visual impediment, deafness or hearing impediment, muteness or speech impediment, and physical reliance on a guide dog, service dog, wheelchair or other remedial appliance or device” [64–67]. Other provinces and territories use slight variations on this definition, and courts and tribunals interpreting the term have given it a broad purposive definition. Whatever minor variances exist in the precise wording or definition of physical disability among the various jurisdictions are likely immaterial in the context of determining whether a particular allergy will be defined as a disability. In most cases of allergy it would be difficult to argue that the condition did not constitute a physical disability protected by human rights law, although it is possible that mild allergic responses may not be covered.

As HRT’s often are mandated to encourage voluntary settlement, and because those settlements are generally confidential, most human right complaints are resolved before they result in a written decision. As a result, there are only a handful of cases assessing the role of allergy under human rights legislation. Nonetheless, the cases that do exist, as well as the broader treatments of discrimination and its justification under human rights law, guide how an HRT will approach questions involving allergy-related human rights complaints in educational contexts and otherwise [56, 68–70].

The question of whether a particular policy discriminates against an individual suffering from an allergy will depend on whether the tribunal accepts that the individual has an allergy sufficient to trigger protection as a disability under the relevant legislation, and, secondly, whether the particular policy complained of has caused a disadvantage to that individual on the basis of that disability. Given the broad mandate of human rights legislation to promote protection of human dignity and rights, and given the broad and inclusive definition of disability outlined above, it is likely that allergies beyond the trivial will constitute disabilities for the purposes of human rights legislation. Certainly, disability raising to the level of anaphylaxis will be found to be a disability as several human rights cases emerging from Ontario have recognized [71–73]. Moreover, in British Columbia, there is a long history of treating allergy as a disability, including environmental irritants such as sensitivity to second hand smoke [74–78].

The issue of whether specific allergy policies *discriminate* on the basis of disability, however, is clouded by greater uncertainty. Under human rights legislation, complainants bear the burden of demonstrating that a rule, policy, or decision constitutes *prima facie* discrimination. As the SCC has held, “to demonstrate *prima facie* discrimination, complainants are required to show that they have a characteristic protected from discrimination under the code; that they experienced an adverse impact with respect to the service; and that the protected characteristic was a factor in the adverse impact” [79]. If potential discrimination is established, “the onus shifts to the defendant to prove on a balance of probabilities that the discriminatory standard […] has a bona fide and reasonable justification” [80].

In *F.T. v Hamilton*, an adjudicator under Ontario’s *Human Rights Code* held that the City of Hamilton’s refusal to ban peanut products at all of the city’s recreational facilities did not discriminate against an individual with allergies [56]. The claimant could not demonstrate that the policy restricted her equal access to the facilities, since the evidence demonstrated that she attended many other venues with potential exposure to peanut products. Moreover, the expert medical evidence tendered in the hearing did not demonstrate that a prohibition on peanuts actually made allergy sufferers safer.

### The duty to accommodate, undue hardship and competing rights

If the rights claimant is able to demonstrate *prima facie* discrimination, the institution or individual responsible for the discrimination must demonstrate that the challenged rule, policy, or conduct was justified. The justification analysis asks whether the rule, policy, or conduct: (1) was adopted for a purpose or goal that is rationally connected to the function being performed; (2) was adopted in good faith, in the belief that it is necessary for the fulfilment of the purpose or goal; and (3) is reasonably necessary to accomplish its purpose or goal, in the sense that it is impossible to accommodate the claimant without undue hardship [80–83].

Most cases turn on the third step of the analysis, and, in particular, on whether the institution or individual has met their obligations to accommodate the rights claimant to the point of undue hardship [84]. An analysis of whether the point of undue hardship has been reached typically considers (as per the OHRT):

- whether the accommodation provider investigated and considered alternative approaches that do not have a discriminatory effect;
- reasons why viable alternatives were not put in place;
- the ability to have differing standards that reflect group or individual differences and capabilities;
- whether the accommodation provider can meet their legitimate objectives in a less discriminatory way;
- whether the standard is properly designed to make sure the desired qualification is met without placing undue burden on the people it applies to; and
- whether other parties who are obliged to assist in the search for accommodation have fulfilled their roles [81, 82].

Institutions can be exempted from a duty to accommodate if they can show that accommodation of the individual rights of the claimant would impose undue hardship. The SCC has held that “the various factors (in assessing undue hardship) are not entrenched, except to the extent that they are expressly included or excluded by statute” [83]. In general, courts and tribunals focus on issues concerning both the process (were alternatives considered?) and substance of accommodation (how practical, feasible, and costly are the proposed alternatives?). In Ontario, the Code requires three considerations in determining whether the threshold of undue hardship has been reached: cost that would alter the essential nature of the enterprise or substantially affect its viability; outside sources of funding such as government programs; and ability to fulfill health and safety requirements [65, 85]. Other provinces and territories use slightly different language but undertake a similar analysis [61–64, 66, 67, 86–88].

There is no general right for children to have specific foods of their parents’ choosing at school, unless those foods are themselves necessary for medical purposes, or, relate to a sincerely held religious practice. For example, in 2014 the BC HRT found that a school with a peanut ban that refused to enroll a diabetic child who had peanut butter in his emergency response kit was liable for failing to consider alternative approaches, and for being unable to demonstrate that denying enrolment “was reasonably necessary to accomplish the broader goal of protecting other students from exposure to peanuts” [89]. Outside of medically necessary foods, it is unlikely that any child advocacy group could establish a strong legal argument that a food ban infringes on the rights of the majority of students.

Accordingly, human rights and constitutional law likely require schools and educational institutions to consider and implement appropriate policy responses to dealing with the disabilities of allergy sufferers. Appropriate policy responses, however, exist along a broad spectrum of possibilities: from individualized protections for specific allergy sufferers (maintaining allergen-free spaces, emergency preparedness) to partial or total food bans. All of this suggests a reasonably high degree of flexibility for institutions in crafting their particular policies in relation to allergies. It seems unlikely that stringent food bans would be found to discriminate against those without allergies, so long as the institution accommodated individuals with a particular medical or religious need for the banned food. At the same time, given the evidence that total bans may not, in fact, increase safety for allergy sufferers [10, 11], it also seems likely that allergy policies instituting something less than a complete ban but with contingencies to deal with emergencies may also be lawful. The best policy advice for institutions seeking to craft sensible, effective, and rights sensitive policies around allergies is to consider the particular contextual facts of their setting and follow the dictates of the best available scientific research on best practices for allergy safety and management. In other words, there is no need for a one definitive model to deal with allergies, but rather the development of particular policies attendant to the circumstances of particular institutional settings and informed by evidence-driven risk assessment. At the moment, the bulk of that evidence appears to point to the appropriateness of total bans for young children in day cares and kindergarten where food sharing will be difficult to control, [55] and something more nuanced when dealing with older populations of students or members of the general public [10, 11].

## Conclusion

It is clear that in most if not all provinces, allergy is legally a physical disability and, as a result, there are obligations to accommodate in the context of schools. We can reasonably conclude based upon the jurisprudence that anaphylaxis-level allergy constitutes a disability under both the *Charter* and human rights legislation, despite the fact that higher courts have not definitively ruled on the matter. Accordingly, schools must make careful decisions about how to deal with life-threatening allergies among their students. Ultimately, if bans can be shown to be effective, they may be a convenient, reasonably simple, and inexpensive way for schools to create clear boundaries and a safe environment for children. Budgetary considerations, fear of litigation, and the social climate can all lead school authorities to ban foods. Ironically, some evidence suggests that bans may be counter-productive and undermine the purposes they aim to serve. In our view, avoiding total bans is, in most cases, in the best long-term interest of all parties, including allergic students. Important protections for allergy sufferers and the school community will continue to include education about food sharing, vigilance, and adequate emergency response mechanisms [12].

The issue of dealing with allergies in schools is not going to disappear. Schools have a duty to keep their students safe. Human rights and constitutional law further require the creation of a learning environment in which the rights of individuals with disabilities are considered and accommodated in a manner that respects everyone’s rights to equal participation. The law creates the overall structure and guiding principles in which our educational institutions must operate, but it is the medical advice of experts and their evidence-based research that hold the key to the content of the policies that schools should adopt.
